# Supplementary material for: Sensitivity of simulated ammonia fluxes in Rocky Mountain National Park to measurement time resolution and meteorological inputs
Source: Atmos Chem Phys. Author manuscript; Available in PMC 2026 Mar 11. (PMC12973231; doi:10.5194/acp-25-15245-2025)
Supplement: Supplement1 [file NIHMS2126066-supplement-Supplement1.pdf]

Supplement of Atmos. Chem. Phys., 25, 15245–15261, 2025
https://doi.org/10.5194/acp-25-15245-2025-supplement
© Author(s) 2025. CC BY 4.0 License.

*Supplement of*

# Sensitivity of simulated ammonia fluxes in Rocky Mountain National Park to measurement time resolution and meteorological inputs

**Lillian E. Naimie et al.**

*Correspondence to:* Jeffrey L. Collett Jr. (collett@colostate.edu)

The copyright of individual parts of the supplement might differ from the article licence.

**Section S1**

AirSentry $NH_3$ concentration measurements at the nearby EPA shelter are compared with mean biweekly passive $NH_3$ data. The biweekly passive $NH_3$ values are a weighted average of all tower-top passive measurements made during the 2-week period. Note, the AirSentry $NH_3$ measurements were taken at the nearby EPA shelter, at a height of 2 m above a grassland ecosystem, and the passive $NH_3$ measurements were made at a height of 25 m above a forest ecosystem on the NEON tower. AirSentry $NH_3$ concentration measurements at the nearby EPA shelter are compared with University Research Glassware (URG) denuder measurements taken on the tower during the summer of 2021. URG annular denuder and filter pack samplers were deployed at the site to measure inorganic gas and particle species [Allegrini et al., 1987; Allegrini et al., 1994; Fitz, 2021]. For this work, we will only consider gas-phase $NH_3$ measurements. Measurements were conducted as described in Naimie et al. [2022], with a few notable differences. The flow was increased from 10 L $min^{-1}$ to 16 L $min^{-1}$ to decrease the sampling time needed. Samples were collected for 4-hour periods during the day and 6 hours overnight. Samples were collected in duplicate at the tower top (25.35 m-agl) and a mid-canopy height. Only the tower top samples are considered for this work. The injection volume of both methods was increased from 50 µL to 200 µL and the analysis time was 17 minutes. Based on previous work the relative standard deviations (RSDs) of major aerosol ion concentration measurements ($NO_3^-$, $SO_4^{2-}$, and $NH_4^+$) are estimated to be between 3-5% and the RSDs for replicate denuder gas concentration measurements are estimated to be approximately 10% [Lee et al., 2004].

In Fig. S1, raw AirSentry $NH_3$ data (panel b.) and AirSentry $NH_3$ data normalized to the biweekly passive concentration (panel c.) are plotted against the URG $NH_3$ data. Generation of the 30-minute $NH_3$ data set (panel c.) is described in section 2.7 (Data Preparation) of the main text. URG sample periods were 4 hours during the day or 6 hours overnight. On sampling days, 2 or 3 daytime samples were taken, and 1 overnight sample was taken. In both panels, the 95% confidence interval of the linear fit (shown shaded in red) overlaps with the one-to-one line (y = x). Here we note that the concentrations during the intensive sampling period agree well between the AirSentry and URG measurements of $NH_3$. Additionally, the normalized AirSentry data set (panel b.), which will be used for the bidirectional exchange simulations, agrees well with the URG measurements of $NH_3$.

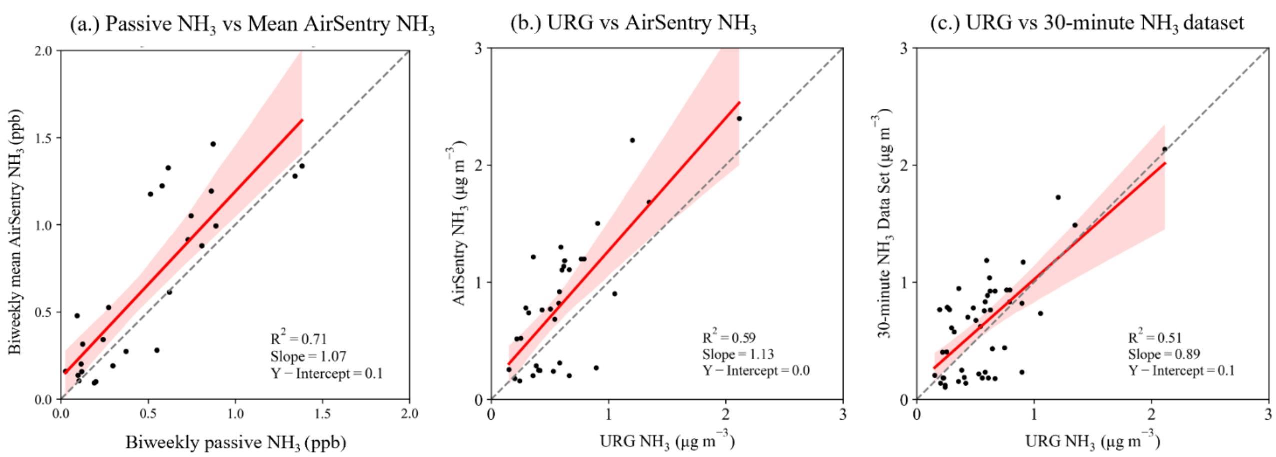


**Figure S1. $NH_3$ measurements from the nearby EPA shelter using an AirSentry are compared with biweekly passive and URG denuder measurements taken at the NEON tower. The least-squares linear fits are shown in red, with the 95% confidence interval shaded for the fit. The one-to-one line is grey-dashed. (a.) $NH_3$ concentration data from the AirSentry, averaged to a 2-week mean, are compared directly to mean biweekly passive $NH_3$ data. (b.) $NH_3$ concentration data from the AirSentry are compared directly to URG denuder measurements. (c.) The scaled AirSentry 30-minute $NH_3$ data was generated using the AirSentry $NH_3$ and biweekly passive $NH_3$.**

## Section S2

Parameters used for the bidirectional exchange simulations are compared between the ERA5 reanalysis and NEON tower in situ data. Units for each parameter are given in the title. We find that temperature (e.), pressure (f.), air density (g.), and soil temperature (i.) agree very well. Each has an $R^2$ above 0.8 and a slope within 10% of unity. Notably, Obukhov length (c.) from ERA5 reanalysis and NEON tower in situ data are not correlated. The other parameters important for understanding the turbulent nature of the atmosphere (wind speed, and friction velocity) have some correlation between data sets.

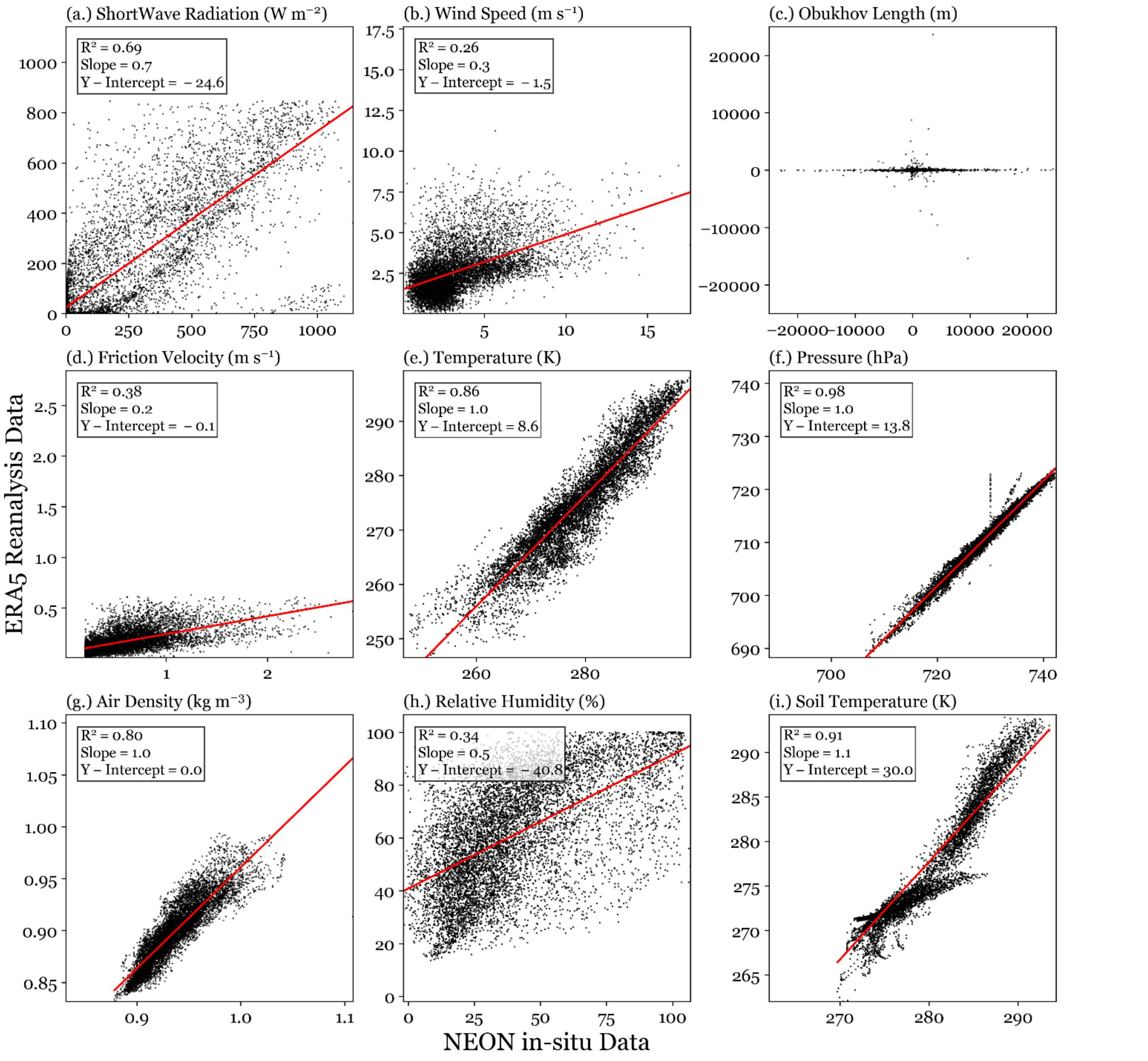


**Figure S2. NEON in-situ measurement data is plotted against the ERA5 reanalysis data for each parameter used in the bidirectional exchange simulations. The linear fit for each pair of data is given in red.**

**Section S3**

$NH_3$ mixing ratios were used to generate an annual average diel pattern. The variability of mixing ratios at 30-minute time steps is shown in Fig. S3a. The mean diel profile is shown in Fig. S3b.

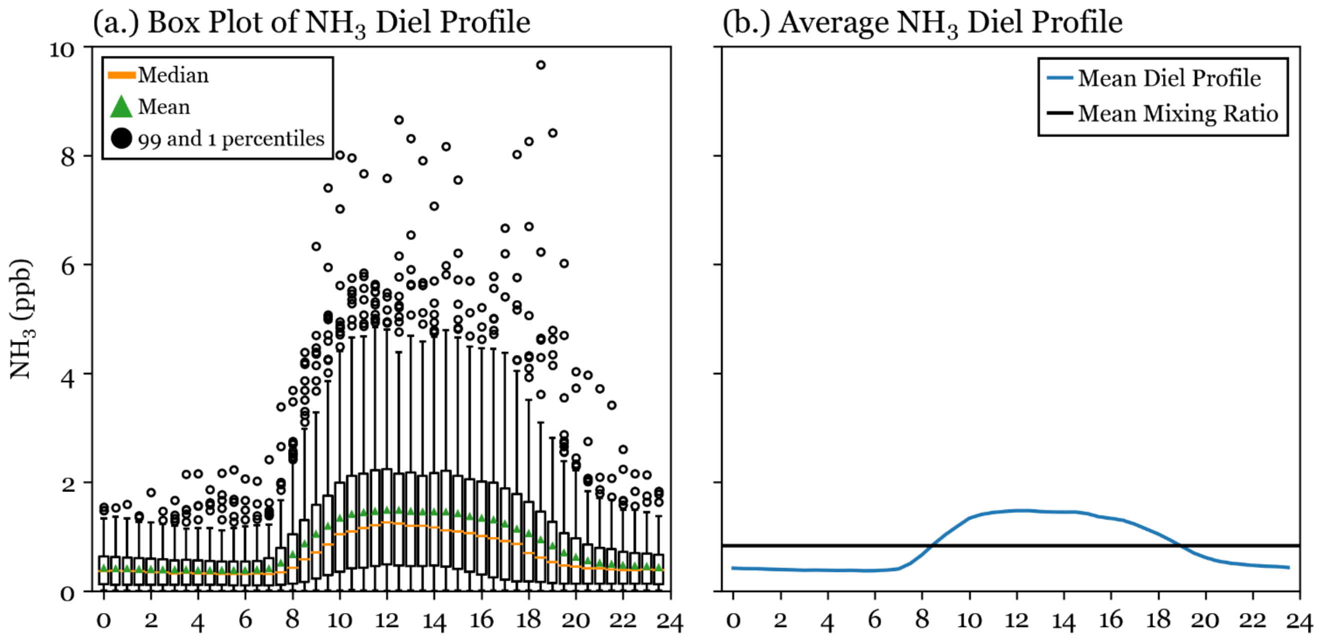


**Figure S3. The diel pattern of the $NH_3$ mixing ratio is shown for the full year of data. (a.) Box plot of 30-minute $NH_3$ mixing ratio values. The median of each box is shown in orange, the mean is shown as a green triangle, the boxes show the 25$^{th}$ and 75$^{th}$ percentile, the whiskers are determined at 1.5 times the interquartile range, and the black dots show outliers. (b.) The mean diel profile is shown in green, and the annual average mixing ratio is black.**

**Section S4**

Figure S4 shows a comparison of the 30-minute $NH_3$ and biweekly $NH_3$ simulations. The 30-minute $NH_3$ simulation uses in situ meteorology. The biweekly $NH_3$ simulation has two sets of simulations run with biweekly integrated $NH_3$ concentration: 1. Simulated with in situ meteorology and 2. Simulated with ERA5 meteorology.

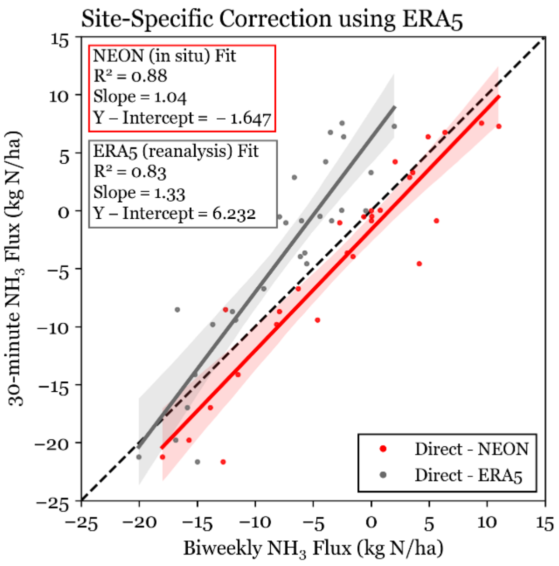


**Figure S4. Bidirectional $NH_3$ flux simulations are plotted for 30-minute $NH_3$ concentration data and biweekly integrated $NH_3$ concentration data. The biweekly $NH_3$ simulations were run using in situ (NEON Tower) and reanalysis (ERA5) meteorology and soil parameters. Bidirectional $NH_3$ fluxes are plotted as net flux over a two-week period. The least squares linear regression is plotted for the data. Shading around the linear regression shows the 95% confidence interval of the fit.**

## Section S5

The sensitivity of our flux results was tested against several key parameters: LAI, TAN, $NH_3$ concentration, and ground emissions. NEON derives LAI at 1 m resolution using remote sensing data. The 1 km by 1 km grid cell surrounding the NEON tower site in RMNP in shown in Fig S5. The box used to estimate a mean LAI value for the area surrounding the site was generated to maximize the area covered without intersecting with roadways or buildings near the site.

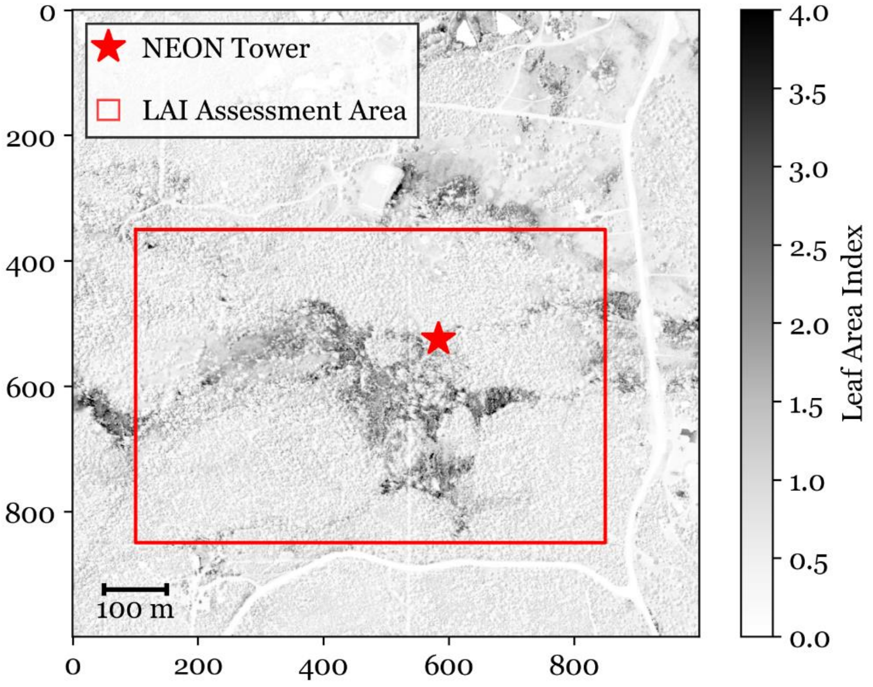


**Figure S5. Leaf area index is shown from the NEON product: "LAI-spectrometer-mosaic". The axis values are meters of the given grid cell from the product output of 1 km by 1 km land surfaces.**

For sensitivity testing, a set of mean values was generated for average square areas beginning at 1 square meter and increasing to 400 square meters around the tower site. The minimum value was 0.2 and the maximum value was 1.4. These are likely extreme values. The response of simulations to LAI changes is shown in Fig. S6a. On average, reducing LAI to 0.2 made $NH_3$ fluxes more positive by 0.08 ng N $m^{-2}$ $s^{-1}$ and increasing LAI to 1.4 made $NH_3$ fluxes more negative by 0.09 ng N $m^{-2}$ $s^{-1}$. The sensitivity to TAN values was assessed using the standard deviation (4.7) of measurements taken by Stratton et al. (2018). In Fig. S6b., flux simulations using the mean TAN value measured by Stratton et al. (2018) is compared with simulations using a TAN value of 5.9 and 15.3 mg $kg^{-1}$. $NH_3$ fluxes shown here are more sensitive to the change in TAN than LAI. On average, decreasing TAN to 5.9 mg $kg^{-1}$ made $NH_3$ fluxes more negative by 0.9 ng N $m^{-2}$ $s^{-1}$ and increasing TAN to 15.3 made $NH_3$ fluxes more positive by 0.9 ng N $m^{-2}$ $s^{-1}$.

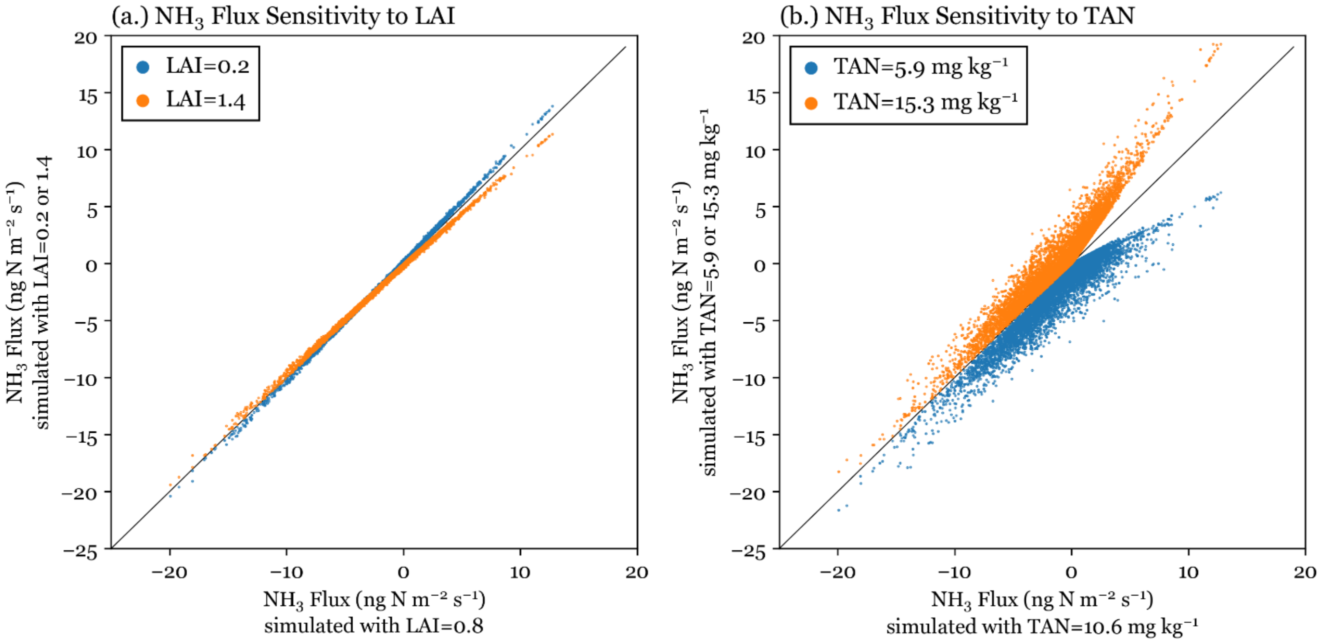


**Figure S6. $NH_3$ flux sensitivities are assessed for (a.) LAI and (b.) TAN. Each sensitivity test is plotted against the initial simulation parameters (LAI=0.8 and TAN=10.6 mg kg$^{-1}$).**

We tested the sensitivity of $NH_3$ flux to changes in atmospheric $NH_3$ concentration. The atmospheric $NH_3$ concentration was scaled by values ranging from 0.5 to 1.5. The observed bias for passive $NH_3$ sampling methods from Puchalski et al. (2011) was a 9% underestimation. Increasing (decreasing) $NH_3$ concentration by 10% increased (decreased) the seasonal mean fluxes by 0.1 to 0.6 ng N m$^{-2}$ s$^{-1}$.

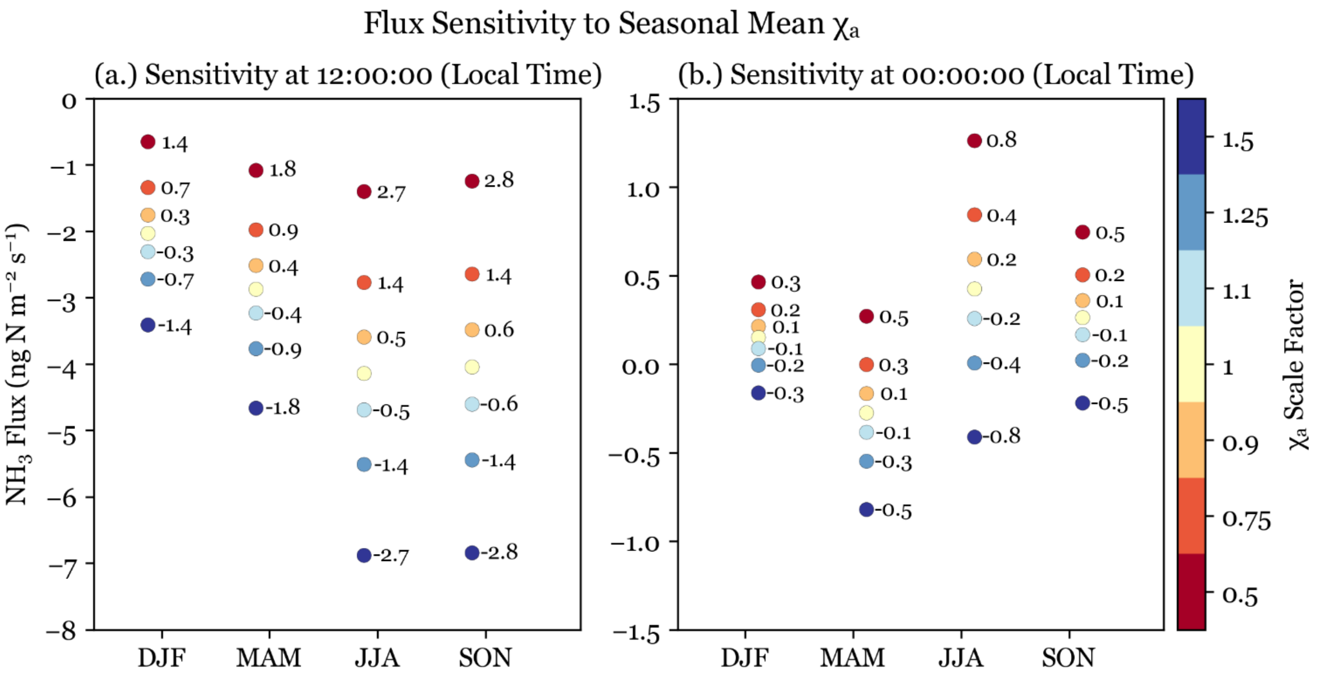


**Figure S7. $NH_3$ flux was simulated using Massad et al. (2010) using the mean seasonal meteorological parameters, mean seasonal $NH_3$ concentration at (a.) 12:00:00 and (b.) 00:00:00, and scaled $NH_3$ concentrations. Points are colored by scale factor applied to the $NH_3$ concentration. Colors are shown in the right hand justified colorbar. Difference to the mean concentration simulation values are listed next to each point (ng N $m^{-2}$ $s^{-1}$).**

At both times (12:00:00 and 00:00:00) the differences in simulated fluxes are uniformly distributed for the reduced and increased values. Notably at 12:00, when all mean simulated fluxes are negative, the relative changes are similar across seasons and close to the input scale factor. At 00:00, when mean fluxes are closer to zero, the fractional differences are much larger. We also see that flux direction changes for these simulations with a concentration change of only about 25%.

The absolute difference in fluxes is typically, although not always, larger for the larger magnitude mean fluxes. While the largest spread at 00:00:00 is about 1.75 (ng N $m^{-2}$ $s^{-1}$). Interestingly in the night simulation (00:00:00), the difference between minimum and maximum simulated values is largest for JJA, which is not the largest magnitude flux value.

$NH_3$ concentration sensitivity was also tested by scaling the input concentration by 9% to account for the error discussed in Puchalski et al. (2011). This resulted in an annual deposition increase of 47%, indicating the importance of accurate $NH_3$ measurements for flux modelling.

Lastly, we tested the sensitivity of flux simulations to the ground compensation point during the winter (December, January, and February), to probe the potential impact of snow cover. In the winter, we set $\chi_g$ to zero to stop ground emissions. Setting $\chi_g$ to zero during winter changed the net wintertime flux from emission to deposition and increased the annual $NH_3$ deposition by 0.06 kg N $ha^{-1}$.

**Section S6**

We conducted two case studies to probe the importance of u* and Obukhov Length on simulated aerodynamic resistances. In case study 1 (2), friction velocity (Obukhov Length) from ERA5 was replaced with the value from NEON (see Fig S8.). This isolates the impact on $R_a$ from each of the given parameters. In Fig S8a and b, we see that the relative impacts of Obukhov Length and u* on $R_a$ aresubstantial. Considering the simulated fluxes in Fig S8c. we see that flux simulations with ERA5 meteorology inputs are more sensitive to changes in $u_*$ than L. In Fig S8a. calculated $R_a$ values are all below 30 s $m^{-1}$ when using $u_*$ from NEON. This is likely due to the minimum $u_*$ value of 0.2 set by NEON.

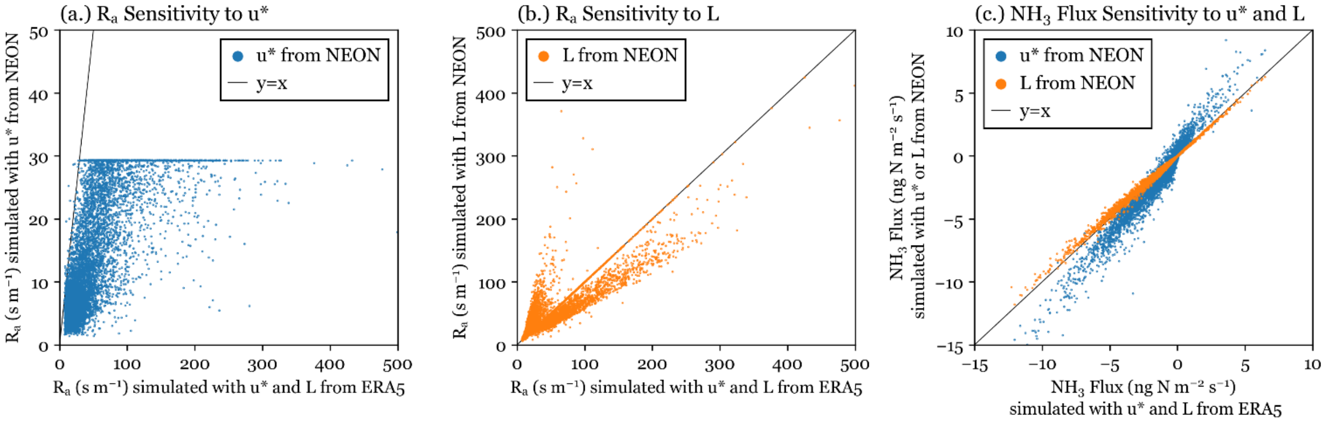


**Figure S8. $R_a$ simulations using ERA5 meteorology are compared to $R_a$ where (a.) u* and (b.) Obukhov Length, have been replaced with in situ values from NEON. (c.) $NH_3$ fluxes are compared where $u_*$ and L have been replaced with in situ values from NEON.**

## Section S7

Measurements of foliage from the NEON site were used to determine the stomatal emission potential. At the NEON site, due to experimental constraints, foliage was collected in locations adjacent to the NEON tower footprint by CSU staff. Five trees were identified for each of the three primary overstory and understory species, for a total of 15 trees. Foliage samples were collected seasonally beginning in the summer of 2021. During each sampling event, 30 g of needles or leaves were collected from each tree by hand, where accessible (preferred) or using a slingshot method to collect a small branch from overhead. Samples were stored in Ziploc bags and shipped overnight to the EPA laboratory in a cooler with ice packs.

Litter and foliage samples were processed and analyzed for pH and $NH_4^+$ by the EPA as follows. 4.0 g of fresh material is subsampled from the plot-level composite sample, ground in liquid nitrogen using a mortar and pestle and small coffee grinder, then extracted with 40 mL of deionized water. pH is determined directly on the extracts. [$NH_4^+$] in the extracts, which reflects the bulk tissue concentration, is determined by ion chromatography after separation of the $NH_4^+$ from the solution as NH3 using headspace equilibration. For the headspace method, 5 mL of tissue extract is added to a 250 mL high-density polyethylene jar containing two ALPHA passive samplers (Center for Ecology and Hydrology; Tang et al., 2001), without the diffusion barrier, affixed to the interior of the lid. The jar is sealed, and 5 mL of 0.3 N NaOH is added to the extract via septum.

$NH_3$ liberated from the liquid extract into the headspace is collected by the passive diffusion samplers over a period of 48 hours, after which the passive sampler is extracted with 10 mL of deionized water. $NH_4^+$ in the extracts is determined by ion chromatography.

To determine the stomatal emission potential, the weighted mean of $\Gamma_{st}$ was calculated using the percentages of evergreen forest and deciduous forest from the NEON site survey (NEON, 2025). The resulting $\Gamma_{st}$ was 29.

## References

Allegrini, I., F. De Santis, V. Di Palo, A. Febo, C. Perrino, M. Possanzini, and Liberti, A.: Annular denuder method for sampling reactive gases and aerosols in the atmosphere, *Science of The Total Environment*, 67(1), 1-16, doi:https://doi.org/10.1016/0048-9697(87)90062-3, 1987.

Allegrini, I., A. Febo, C. Perrino, and Masia, P.: Measurement of Atmospheric Nitric Acid in Gas Phase and Nitrate in Particulate Matter by means of Annular Denuders, *International Journal of Environmental Analytical Chemistry,* 54(3), 183-201, doi:10.1080/03067319408034088, 1994.

Benedict, K. B., C. M. Carrico, S. M. Kreidenweis, B. Schichtel, W. C. Malm, and Collett, J. L.: A seasonal nitrogen deposition budget for Rocky Mountain National Park, *Ecological Applications*, 23(5), 1156-1169, 2013.

Fitz, D.: Evaluation of diffusion denuder coatings for removing acid gases from ambient air. U.S. Environmental Protection Agency, Office of Air Quality Planning and Standards, Research Triangle Park, NC. http://www.epa.gov/ttnamti1/files/ambient/pm25/spec/denudr.pdf(open in a new window), 2002.

Lee, T., S. M. Kreidenweis, and Collett, J. L.: Aerosol Ion Characteristics During the Big Bend Regional Aerosol and Visibility Observational Study, *Journal of the Air & Waste Management Association,* 54(5), 585-592, doi:10.1080/10473289.2004.10470927, 2004.

Lee, T., X.-Y. Yu, B. Ayres, S. M. Kreidenweis, W. C. Malm, and Collett, J. L.: Observations of fine and coarse particle nitrate at several rural locations in the United States, *Atmospheric Environment,* 42(11), 2720-2732, doi:https://doi.org/10.1016/j.atmosenv.2007.05.016, 2008.

Naimie, L. E., A. P. Sullivan, K. B. Benedict, A. J. Prenni, B. C. Sive, B. A. Schichtel, E. V. Fischer, I. Pollack, and Collett, J. L.: PM2.5 in Carlsbad Caverns National Park: Composition, sources, and visibility impacts*, Journal of the Air & Waste Management Association*, 72(11), 1201-1218, doi:10.1080/10962247.2022.2081634, 2022.

National Atmospheric Deposition Program (NRSP-3). NADP Program Office, Wisconsin State Laboratory of Hygiene, 465 Henry Mall, Madison, WI 53706, 2022.

NEON (National Ecological Observatory Network). Terrestrial Observation System (TOS) Site Characterization Report: Domain 10. NEON.DOC.003883vB. Dataset accessed from https://data.neonscience.org/documents/-/document_library_display/kV4WWrbEEM2s/view_file/2263497 on June 19, 2025.

Puchalski, M. A., M. E. Sather, J. T. Walker, C. M. B. Lehmann, D. A. Gay, J. Mathew, and Robarge, W. P.: Passive ammonia monitoring in the United States: Comparing three different sampling devices, *Journal of Environmental Monitoring,* 13(11), 3156-3167, doi:10.1039/C1EM10553A, 2011.

Schrader, F., M. Schaap, U. Zöll, R. Kranenburg, and Brümmer, C.: The hidden cost of using low-resolution concentration data in the estimation of $NH_3$ dry deposition fluxes, *Scientific Reports,* 8(1), 969, doi:10.1038/s41598-017-18021-6, 2018.

Yu, X.-Y., T. Lee, B. Ayres, S. M. Kreidenweis, J. L. Collett, and Malm, W.: Particulate Nitrate Measurement Using Nylon Filters, *Journal of the Air & Waste Management Association,* 55(8), 1100-1110, doi:10.1080/10473289.2005.10464721, 2005.
